# Effect of combined contraceptive pill on immune cell of ovarian endometriotic tissue

**DOI:** 10.1186/s13048-021-00819-8

**Published:** 2021-05-12

**Authors:** Wanwisa Waiyaput, Keerati Wattanakamolchai, Yada Tingthanatikul, Srithean Lertvikool, Siriluk Tantanavipas, Kanthanadon Dittharot, Morakot Sroyraya, Areepan Sophonsritsuk

**Affiliations:** 1 Office of Research Academic and Innovation, Faculty of Medicine Ramathibodi Hospital, Mahidol University, 10400 Bangkok, Thailand; 2 Department of Obstetrics and Gynecology, Faculty of Medicine, Khon Kaen University, 40002 Khon Kaen, Thailand; 3 Reproductive Endocrinology and Infertility Unit, Department of Obstetrics and Gynaecology, Faculty of Medicine Ramathibodi Hospital, Mahidol University, 10400 Bangkok, Thailand; 4 Department of Anatomy, Faculty of Science, Mahidol University, 10400 Bangkok, Thailand

**Keywords:** Combined contraceptive, Endometriosis, Immune cells, Macrophage, Natural killer cell, Regulatory T-cell

## Abstract

**Background:**

Dysregulation of immune response is associated with development of endometriosis. The study aim was to evaluate effect of combined oral contraceptive pills (COCs) consisting of ethinyl estradiol (EE) and desogestrel on the expression of macrophage, natural killer cells, and regulatory T cells of ovarian endometriotic cysts.

**Methods:**

Endometriotic cyst wall tissues were collected from women with endometriosis who were treated (n = 22) with COCs (one table per day of EE 0.03 mg and desogestrel 0.15 mg administered for 28 to 35 days before surgery) or untreated (n = 22). The tissues were collected from endometriotic cyst wall during laparoscopic or laparotomy ovarian cystectomy. Immunohistochemistry for anti-CD68, anti-CD56, and anti-forkhead–winged helix transcription factor (FoxP3), a marker for macrophages, natural killer cells, and regulatory T cells, respectively, were investigated.

**Results:**

The median (interquartile range [IQR]) number of anti-CD68 positive cells in the COC group was significantly lower than in the untreated group (12.7; 4.9–19.3) versus 45.7 (26.0–70.7), p < 0.001). Tissue infiltration of anti-CD56 positive cells in endometriotic cyst was significantly higher after the treatment when compared with tissue from untreated group (42.9, 27.4–68.9 versus 25.3 (14.1–37.3; p = 0.009). The number of regulatory T cells was also significantly increased in the COC group (6.3, 2.8–15.5) versus 0 (0–1.8; p < 0.001).

**Conclusions:**

The effects of COC, containing EE 0.30 mg with desogestrel 0.15 mg, on the immune system was demonstrated by a significant decrease in the number of macrophages and an increase in natural killer and regulatory T cells.

## Introduction

Endometriosis is diagnosed when endometrial tissue grows outside the uterus. Sampson’s theory, one of many proposed theories, explained that the disease originates from the reversal of blood flow during the menstrual cycle to the pelvic cavity [1]. The defective immunity is another frequently-mentioned pathogenesis of this disease in addition to other factors, including coelomic metaplasia, Mullerian rests, lymphatic and hematogeneous spread, and differentiated stem cells [2].

A dysregulated immune system could cause endometriosis initiation and progression. The retrograde endometrial tissues during menstruation that escape from clearance by the immune response could attach and invade the pelvic cavity structures. The ectopic endometrium stimulates inflammatory responses leading to secretion of various cytokines in tissue, peritoneal fluid (PF), and serum. Many studies demonstrate a significant increase in number of macrophages in the eutopic endometrium and PF during all stages of endometriosis [3–6]. The numerous macrophages in peritoneal fluid of patients with endometriosis secrete many kinds of enhancing cytokines for endometriosis progression, including pro-inflammatory cytokines [7]; tumor growth factor (TGF)-β [8], interleukins (IL)-6 and-10, angiogenic factors, vascular endothelial growth factor (VEGF) [9, 10], and an inhibitory cytokine; IL-24 [11]. The polarization of alternative (M2) macrophages from classic (M1) macrophages was shown in endometriotic cells [12]. M2 macrophages secrete higher levels of pro-inflammatory cytokines than M1 macrophages [13].

Uterine natural killer (NK) cells are the predominant leukocytes in normal endometrium and responsible for host rejection of tumors or infected cells. NK cells are likely to be involved in immune responses in endometriosis demonstrated by a reduction in NK cell cytotoxicity in the peripheral blood, peritoneum, and PF [14]. The cytotoxicity of NK cells derived from women with endometriosis is reduced as demonstrated by a reduction in the natural cytotoxicity receptor, NKp46 [15], and a cell surface marker for cytotoxicity, CK107a [16]. However, the inhibitory cytotoxic receptor, CD94/NKG2A, significantly increases in peritoneal NK cells [17]. Regulatory T cell (Treg), identified by forkhead–winged helix transcription factor family member (FoxP3), is the key regulator to suppress activation and maintain homeostasis of immune cells, and tolerance to self-antigens. The high Treg number and levels of marker in endometriotic lesions and PF leads to the decreased recruitment of immune cells preventing the targeting of retrograde endometrial cells [18]. However, inconclusive evidence about Treg cell concentration in eutopic endometrium and circulation has been found [19]. The number of Tregs decreased in endometrium and circulation in women without endometriosis between late proliferative and early menstrual secretory phases [20]. However, the number of Tregs in the eutopic endometrium remained significantly high during the entire secretory phase in endometriosis [21]. The higher number of Tregs may reflect the overabundant suppression of activated immune cell in the endometriotic lesion.

Although the cause of endometriosis is unclear, the disease is well-known for its estrogen-dependent disorder [22, 23]. Progestin and combined oral contraceptives (COCs) are the first line treatment. Progestin exerts marked endometrial decidualization or atrophy of both eutopic and ectopic endometrium [24, 25]. COCs are popular among other medications because of their high efficacy, low side effects, and low cost. Use of COCs suppress the release of gonadotropins by negative feedback of estrogen and progestin to gonadotropin, inhibit ovarian function, and cause decidualization of the endometrium. Moreover, COCs also have been shown to cause down-regulation of cell proliferation and lead to an increase apoptosis in the eutopic endometrium of women with endometriosis [26]. Limited information concerning COCs on immune cells of women with endometriosis have been reported; therefore, the present study aimed to investigate the effect of COCs on immune cells of endometriotic tissue.

## Materials and methods

The study was conducted from September 2015 to October 2017 in the Reproductive Endocrinology and Infertility Unit, Department of Obstetrics and Gynaecology, Ramathododi Hospital. The study was approved by the Ethical Clearance Committee on Human Related Research Involving Human Subjects and Faculty of Medicine at Ramathibodi Hospital, Mahidol University (MURA2014/205). Ectopic endometrium (endometriotic cyst wall) samples from 44 reproductive women with endometriosis were obtained from women treated/not treated with COCs. Women in the treated group received one tablet per day of COCs containing ethinyl estradiol (EE) 0.03 mg with desogestrel 0.15 mg for 28 to 35 days before surgery. Women were diagnosed with ovarian endometriotic cysts with sizes equal to or more than 3 cm based on ultrasonography, no previous history of using any oral hormones three months prior to study enrollment, those who did not receive depot-medroxyprogesterone acetate (DMPA) or gonadotropin releasing hormone agonist (GnRH) agonist within nine months prior to study enrollment, and willingness to participate in the study. The exclusion criteria included underlying diseases, such as cirrhosis, coagulopathy, and chronic kidney, heart and pulmonary diseases. The tissues were collected from the endometriotic cyst wall during laparoscopic or laparotomy ovarian cystectomy.

All collected specimens were prepared for formalin-fixed paraffin-embedded tissue blocks for subsequent immunohistochemical studies with monoclonal anti-mouse CD68 antibody (clone PG-M1; DAKO antibody; Denmark) as a marker for macrophages, monoclonal anti-mouse CD56 antibody (clone NK-1; Thermo Fisher Scientific; Waltham, USA) as a marker for NK cells, and monoclonal anti-mouse FoxP3 antibody (236 A/E7; Abcam antibody ab20034; Cambridge, MA) for Tregs. We used UltraVision Quanto Detection System HRP DAB kit, Thermo Scientific as secondary antibody. The number of CD68, CD56, and FoxP3 brown spots were counted in 20 different fields (200 × 200 µ) for each person (× 200 magnification) under microscopy. The number of positive cells were calculated and expressed as the mean or median positive cells per mm^2^. The results in each biopsy specimen were recounted and confirmed by a second observer who did not know the history of the patient.

### Statistical analysis

Statistical analyses were performed using IBM SPSS Statistics for Windows, version 19.0 (Armonk, NY: IBM Corp). For baseline demographics clinical and surgical characteristics, the chi-square or Fisher’s exact test was used for comparison of categorical variables. Student’s t-test was used to evaluate the result in cases in which the data is normally distributed; otherwise, the Mann–Whitney test was used for comparison of continuous variables. Data were presented as mean ± standard deviation (SD).

## Results

**Fig. 1:**
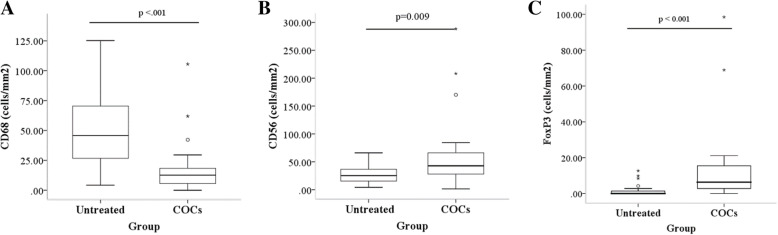
Number of immune cells densities in endometriotic cyst wall tissue compared between treatment and control. **a** number of CD68 positive-stained cells. **b** number of CD56 positive-stained cells. **c** number of FoxP3 positive-stained cells

**Fig. 2:**
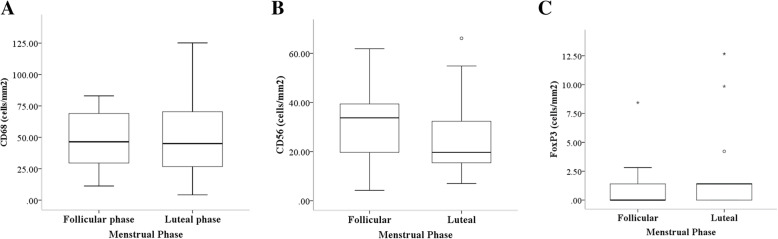
Number of immune cells densities in endometriotic cyst wall tissue compared between follicular and luteal menstrual phases. **a** number of CD68 positive-stained cells. **b** number of CD56 positive-stained cells. **c** number of FoxP3 positive-stained cells

**Fig. 3:**
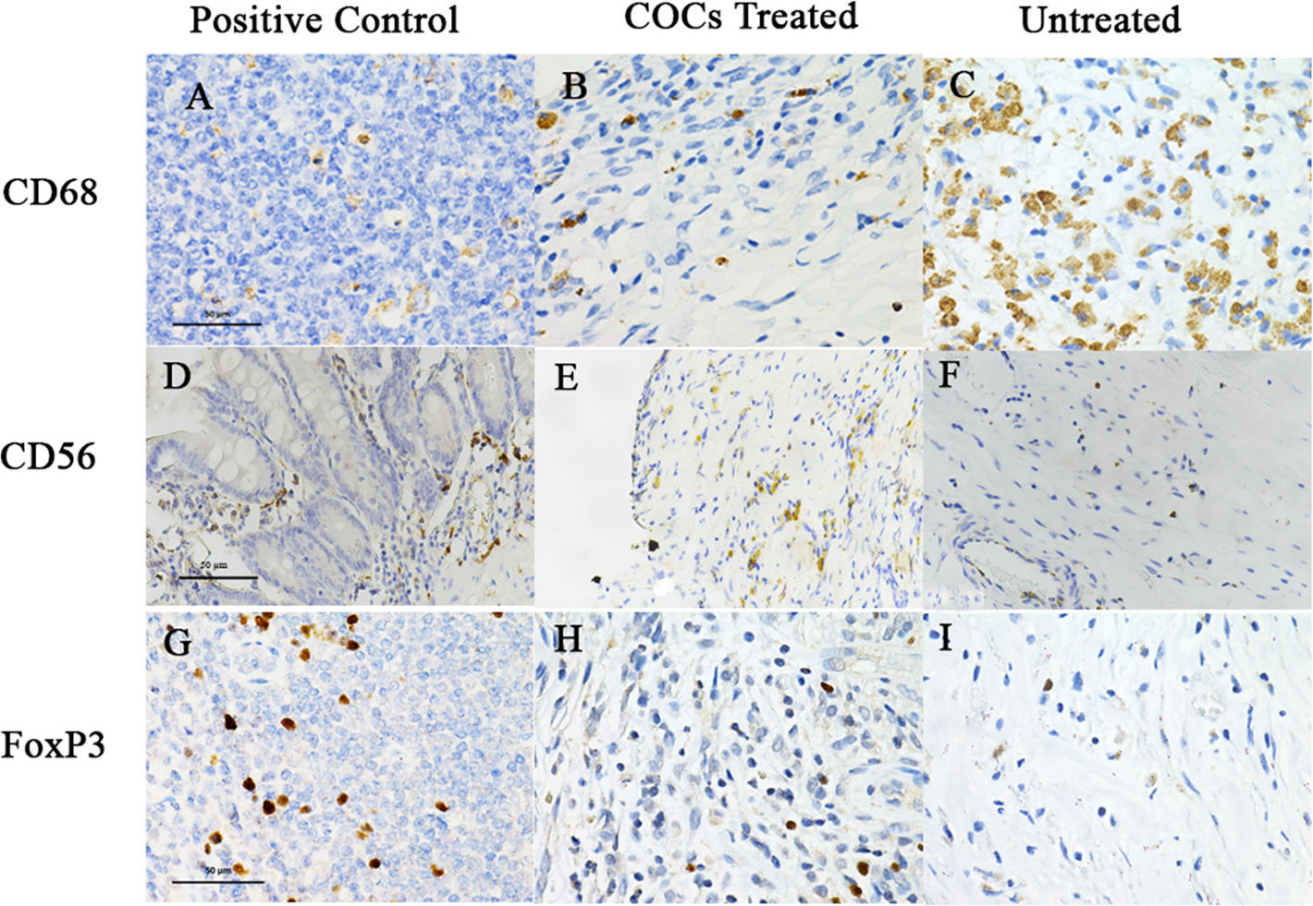
The immunohistochemistry by anti-CD 68, anti-CD56 and anti-FoxP3 in endometriotic cyst wall

No significant differences in age, body mass index, mean diameter of endometriotic cyst, type of surgery (laparoscopy or laparotomy), and American Society of Reproductive Medicine (ASRM) stage of endometriosis between two groups were demonstrated (Table 1). The number of anti-CD68 positive cells in the treatment group was significantly lower than in the non-treatment group (median with IQR 12.7, 4.9–19.3) *versus* 45.7 (26.0–70.7; p < 0.001). Tissue infiltration of anti-CD56 positive cells in endometriotic cyst wall tissues was significantly higher in the treatment group compared with the non-treatment group (median with IQR 42.9, 27.4–68.9) *versus* 25.3 (14.1–37.3; p = 0.009). In addition, the number of Tregs was significantly increased in the treatment group (6.3, 2.8–15.5) *versus* 0 (0–1.8; p < 0.001) as shown in Fig. 1. No significant differences in immune cells between menstrual phases was demonstrated as shown in Figs. 2 and 3.

**Table 1:** Patient characteristics

Characteristics	EE + Desogestrel (N = 22)	Control (N = 22)	*p* value
Age (yrs) (mean ± SD)	30.4 ± 5.1	34.6 ± 5.9	0.11
BMI (kg/m^2^) (mean ± SD)	22.5 ± 4.1	22.2 ± 2.9	0.15
Bilateral (n, %)	6 (27.3 %)	9 (40.9 %)	0.53
Diameter of cyst (cm.) (mean ± SD)	4.7 ± 2.2	6.7 ± 3.6	0.99
Type of operation (n,%) - Laparoscopy - Laparotomy	8 (72.7 %) 3 (27.3 %)	9 (60.0 %) 6 (40.0 %)	0.19
ASRM classification (n,%) - Stage III - Stage IV	6 (54.5 %) 5 (45.5 %)	8 (53.3 %) 7 (46.7 %)	0.55

Note: *BMI* body mass index, *ASRM* American Society of Reproductive Medicine, *EE* e thinyl estradiol

## Discussion

Endometriosis is a complex multi-facet disease. A dysregulated immune response is the emerging known mechanism of disease. In order to investigate the impact of hormonal treatment on defective immunity of endometriosis disease, we compared endometriotic cyst wall tissues from patients treated with COCs containing ethinyl estradiol 0.30 mg/d and desogestrel 0.15 mg/D for 28 to 35 days before surgery to the non-treatment group. The results demonstrated that COCs caused a significant decreased in the number of macrophages and increase in the number of NK cells and Tregs in endometriotic cyst tissues. The tissues were evaluated and quantitated the number of macrophages, NK cells and Treg by immunohistochemistry using anti-CD68, anti-CD56 and anti-FoxP3 antibodies as markers.

Apoptosis or programmed cell death plays a major role in regulating immune homeostasis in peritoneal cavity [27]. Reflux of endometrial cells from menstrual blood into the pelvic cavity induces intense inflammatory responses, which then attracts macrophages and NK cells to get rid of the foreign cells. The reduced apoptosis of mononuclear cells in peritoneal cavity with the increased apoptosis of endometrial cells underlies the peritoneal homeostasis. The dysregulation of the peritoneal homeostasis could cause “immunoescaping” of endometriotic cells. Local estrogen production in combination with an increase in inflammatory response in endometriotic lesions and eutopic endometrium contributes in the abnormal immunologic reaction in endometriosis [28]. Prostaglandins (PGs) and other inflammatory mediators are then generated [29, 30]. Prostaglandin E2 (PGE2) causes activation of the expression of the aromatase gene. Local aromatase enzyme converts androgen to estrogen as demonstrated in eutopic endometrium of patients with endometriosis [31, 32] while absence of aromatase expression in women with no endometriosis. Estrogen then induces a vicious cycle, that is, activating cyclooxygenase (COX)-2 enzyme, resulting in the PG production and increased inflammatory responses. Because of estrogen receptor expression on macrophages, the macrophage responses to local estrogen by reducing its phagocytotic activity to the retrograde endometrial cells [33, 34]. Moreover, macrophages responses to estradiol in the PF of women with endometriosis occurred via *in vivo* of secrete interleukin-6 and tumor necrosis factor-α [35]. Transcription factor nuclear factor (NF)-_K_B is involved in the association of inflammation and aromatase expression. (NF)-_K_B is activated in ectopic lesions and subsequently activates cyclooxygenase (COX-2) and other inflammatory-related genes. NF-_K_B is inactivated by progesterone in endometrium and activated when progesterone withdrawal occurs [36]. Hepatocyte growth factor (HGF) also plays a role in the development of endometriosis. Early and active endometriosis lesions in the peritoneum consist of abundant macrophages that correlate with HGF expressions [4]. PF macrophages are regulated by ovarian steroids with respect to HGF and vascular endothelial growth factor (VEGF) secretion in women with endometriosis [37].

The present study demonstrates that COCs caused a decrease in the number of macrophages and an increase in the number of NK cells in endometriotic lesions. To our knowledge, no study reporting the effect of COCs on macrophages and NK cells in endometriosis has been done. We hypothesize that COCs can modulate these immune cells in many ways. First, COCs could cause a reduction in menstrual bleeding [38, 39] and consequently, menstrual reflux into the pelvic cavity. This reduction would then decrease inflammatory reactions and local estrogen production. The decrease in local inflammation would cause a decrease in macrophage attraction. Second, COCs exert a strong progestogenic effect, which can cause inactivation of both aromatase and COX-2 [40, 41]. Previous studies have demonstrated that COCs can suppress aromatase and COX-2 expression via the strong progestin component that leads to a decrease in local estrogen production and inflammatory responses [30, 40, 41]. This process results in disrupt the vicious cycle of inflammation, local conversion of androgen to estrogen, and the endometriosis development. Third, COCs are well-known for their suppressive effects on hypothalamic–pituitary–gonadal axis and lower estrogen levels [42, 43]; therefore, COCs would provide effects similar to GnRHa. The number of CD68-positive cells and micro-vessel density were found to be significantly decreased in the eutopic endometrium of women with endometriosis and adenomyosis in the GnRH agonist group when compared with that in the non-treated group [44]. Moreover, COCs could possibly regulate macrophages via HGF. Estrogen, more than progesterone, stimulated macrophages to secrete HGF [37]. However, further studies focusing on these mechanisms are required.

The effect of COCs on macrophage polarization in endometriosis is unknown. Macrophage polarization plays a role in inflammatory microenvironment and stage of the endometriotic lesion. M1 macrophages are found in large number in the early stages of endometriosis, stage I-II, whereas the switch to M2 macrophages occurs in stage III-IV.

The early stages of endometriosis are associated with pro-inflammatory state, while the pro-fibrotic activity accounts for the advanced stages [45]. Although data from our study demonstrated that COCs reduce the macrophage number, future study would be needed to explore role of COCs on macrophage plasticity.

The number of NK cells in both peripheral and menstrual blood decreased in addition to the decrease in peripheral and peritoneal NK cytotoxicity in women with endometriosis [46–48]. Our study demonstrates an increase in the number of NK cells in patients with endometriosis and those treated with COCs. Previous data demonstrated the inverse correlation between stage of endometriosis and NKT and NK cells [49]. Therefore, an enhance in NK cell number by COCs could reflect the its protective role on the progression of endometriosis via NKT and NK cells. We did not study the cytotoxic function of NK cells since human NK cells can be divided into at least two subsets (cytotoxic and cytokine-secreting NK cells) [50]. Most NK cells are cytotoxic; therefore, they kill organisms and release low levels of interferon-γ [51]. Future studies would be required to investigate the effect of COCs on NK cytotoxicity.

In this study, we found that COCs caused a significant increase in the number of Tregs when compared with the non-treated group. However, we cannot yet explain the effect of COCs on the causal relationship between Tregs and endometriosis. Data regarding the role of Tregs in endometriosis are scarce. Several studies have demonstrated an increase in Treg number in PF or peripheral blood [19, 52, 53], while the others studies showed the opposite results [54] or no significant difference [55]. Minimal data regarding ovarian steroidal hormones have been reported. Estrogen seems to activate Treg expression and function. Impaired Treg suppression functions were found in the absence of estrogen receptor β in the mice model of intestinal inflammation [56]. Moreover, the number of Tregs correlated with high estrogen levels during pregnancy [56]. Further studies are required to understand their role in endometriosis and hormone effects on Tregs.

The mean number of CD68, CD56, and FoxP3 lymphocytes was not different between the proliferative and secretory phases. Therefore, we compared our combined data between treatment and control group irrespective of menstrual cycle phases. Our results are in line with study of Oosterlynck et al. in which the immunosuppressive effect of peritoneal lymphocytes were studied [57].

### Strengths of the study

This study was the first attempt to examine the impact of COC effects on immune cells, both humoral immunity and cell-mediated immune systems. Most previous studies concerning immune cells investigated PF and peripheral blood, but the present study directly investigated immune cells in ectopic endometrial tissue. The results of this study form the knowledge base for developing new information about mechanism of combined contraceptives in endometriosis.

### Limitations of the study

The outcomes of present trial were obtained from two patient groups. We could not conduct the study in the same patient for comparison between pre- and post-treatment. Moreover, the present trial was an observational study, and it might contain some biases, such as selection bias. Moreover, the period of hormonal therapy was relatively short. A study with a longer hormonal therapy period and conducted as a randomized control trial is needed. A study for the effects of hormone therapy on functional immune cells would be interesting for further research.

## Conclusions

The results of this study showed that COC pills containing ethinyl estradiol 0.30 mg and desogestrel 0.15 mg per tablet affected the immune system by causing a significant decrease in macrophage number and significantly higher NK cells and Tregs, which are not associated with menstrual phase. Our study provided evidence of mechanism of COCs on the endometriotic disease from the immunological aspects.

## Data Availability

The datasets used and/or analyzed during the current study are available from the corresponding author upon reasonable request.
